# Regulating Drug Release Behavior and Kinetics from Matrix Tablets Based on Fine Particle-Sized Ethyl Cellulose Ether Derivatives: An *In Vitro* and *In Vivo* Evaluation

**DOI:** 10.1100/2012/842348

**Published:** 2012-04-29

**Authors:** Kifayat Ullah Shah, Gul Majid Khan

**Affiliations:** Drug Delivery Research Centre, Department of Pharmaceutics, Faculty of Pharmacy, Gomal University D. I. Khan, Pakistan

## Abstract

The design and fabrication of sustained/controlled release dosage forms, employing new excipients capable of extending/controlling the release of drugs from the dosage forms over prolonged periods, has worked well in achieving optimally enhanced therapeutic levels of the drugs. In this sense, the objective of this study was to investigate the suitability of selected cellulose ether derivatives for use in direct compression (DC) and as efficient drug release controlling agents. Controlled release matrix tablets of ciprofloxacin were prepared at different drug-to-polymer (D : P) ratios by direct compression using a fine particle sized ethylcellulose ether derivative (ETHOCEL Standard Premium 7FP) as rate controlling polymer. The tablets obtained were evaluated for various physico-chemical characteristics and *in-vitro* drug release studies were conducted in phosphate buffer (pH 7.4) using PharmaTest dissolution apparatus at constant temperature of 37°C ± 0.1. Similarity factor *f*_2_ was employed to the release profiles of test formulations and were compared with marketed ciprofloxacin conventional tablets. Drug release mechanism and the kinetics involved were investigated by fitting the release profile data to various kinetic models. It was found that with increasing the proportion of ethylcellulose ether derivative in the matrix, the drug release was significantly extended up to 24 hours. The tablets exhibited zero order or nearly zero order drug transport mechanism. *In vivo* drug release performance of the developed controlled release tablets and reference conventional tablets containing ciprofloxacin were determined in rabbit serum according to randomized two-way crossover study design using High Performance Liquid Chromatography. Several bioavailability parameters of both the test tablets and conventional tablets including *C*_max⁡_, *T*_max⁡_ and AUC_0-*t*_ were compared which showed an optimized *C*_max⁡_ and *T*_max⁡_ (*P* < 0.05). A good correlation was obtained between *in vitro* drug release and *in vivo* drug absorption with correlation value (*R* ^2^ = 0.934). Relative bioavailability was found to be 93%. Reproducibility of manufacturing process and accelerated stability of the developed tablets were performed in stability chamber at 40 ± 2°C and 75 ± 5% relative humidity for a period of 6 months and were found to be stable throughout the stability period.

## 1. Introduction

In the current years of development in the field of pharmaceutics, the administration of drugs in a new challenging and controlled approach is being given great attention for improved therapeutic levels. Various controlled release dosage forms have been developed or under development in order to achieve this approach in treating various kinds of diseases because of their improved results and advantages over the conventional dosage forms [1]. An ideal drug delivery system should have two basic properties, the first is the delivery of drug according to the need of the body over a period of treatment, and the second is targeted delivery to the site of action. These properties can be achieved by controlled release drug delivery systems which can improve the therapeutic effect and safety of a drug inside the body by reducing the dosage frequency. Furthermore, the need of cheap medicaments has been generating interest in controlled release drug delivery systems [2]. Various conventional and controlled release dosage forms with different doses have been formulated, and from investigations it is cleared that there is no difference between the conventional dosage forms given several times a day and controlled release dosage form given once a day with respect to bioavailability and therapeutic effects [2].

Drug release is controlled by a combination of several physical processes like diffusion, polymer swallowing, erosion, and dissolution. Swellability of the polymer controls primarily the dissolution and diffusion of the drug by penetration of the water through the tablet. The polymer swellability, erosion control, and the drug release rate, all depend on the molecular weight of the polymer, degree of substitution, and the polymer concentration [1].

Ciprofloxacin is most widely used in UTI's with a good localized action on the infected sites. Ciprofloxacin produces high urine concentrations because about 50% is excreted in urine in unchanged form [3]. It belongs to quinolones which have broad spectrum activity against many types of gram-positive as well as gram-negative bacteria to control various diseases.

Previously we have worked on the development and evaluation of controlled release drug delivery matrices based on different grades and types of polymers [4]. In this paper we have explored design controlled release (CR) matrix tablets of ciprofloxacin HCl testing a fine particle sized ethyl cellulose ether derivative (ETHOCEL Standard Premium 7FP) at different drug-to-polymer (D : P) ratios and the main focus of this study was to investigate influence of different concentration of the polymer and coexcipients on the release rate and mechanism from controlled release tablets.

## 2. Materials and Methods

NaOH (Merck, Germany), mono basic potassium phosphate, ciprofloxacin HCl (Velour Pharma Islamabad, Pakistan), lactose and magnesium stearate (BDH chemical limited, Pool England), ETHOCEL 7P (Dow Chemical Co., Midland USA), acetonitrile HPLC grade (Malinckrodt, USA), methanol (BDH, UK), pentane, dichloromethane (Malinckrodt, USA), ciproxin conventional tabs by Bayar Pharma containing 250 mg ciprofloxacin, HPLC (Perkin Elmer Series 200, USA) Pharma Test Dissolution Apparatus (D-63512, Hainburg), Single Punch Tablet machine (Erweka AR 400, Germany), UV-visible spectrophotometer (UVIDEC-1601 Shimadzu, Japan), friability tester (Erweka TA3R, Germany) and hardness tester (Erweka Apparatus TB24, Germany).

### 2.1. Preparation of Matrix Tablets

#### 2.1.1. Tablet Formulations

Controlled release tablets of ciprofloxacin HCl were formulated for the direct compression method using ETHOCEL 7P polymer at drug-to-polymer ratios of 10 : 1, 10 : 2, 10 : 3, 10 : 4, 10 : 5, 10 : 6, 10 : 7, and 10 : 8 as shown in the Table 1. Lactose was used as filler and magnesium stearate (0.5%) was used as lubricant.

#### 2.1.2. Tablet Compression

For each formulation, a weighed amount of ciprofloxacin HCl along with other excipients was taken and was blended geometrically with the help of pestle and mortar. This mixture was passed through mesh # 8 for three repeated times and a weighed amount of lubricant (Magnesium stearate) was added and was again passed through the same mesh for three repeated times. The mix powder was directly compressed to core tablets each weighing 200 mg by using single punch machine (Erweka AR 400, Germany) using a compaction pressure of 7-8 kg/cm^2^.

### 2.2. Physicochemical Evaluation of CR Tablets of Ciprofloxacin HCl

Ciprofloxacin HCl directly compressed controlled release tablets were evaluated for various physicochemical tests including weight variation, friability, hardness, diameter, and drug content. The average weight of ciprofloxacin HCl tablets were calculated from 20 tablets with the help of digital weighing balance. The hardness test was performed on 10 tablets by using hardness tester (Erweka Apparatus TB24, Germany) and average weight was determined. Friability tests were performed on 20 tablets with the help of friability tester (Erweka TA3R, Germany) at 25 rpm for 4 min and average loss was calculated. The average diameter and thickness was determined by checking the diameter and thickness of 10 tablets by Vernier caliper. For drug content uniformity tests, 5 tablets were randomly collected, pulverized, and weight equivalent to 60 mg ciprofloxacin was extracted by using 100 mL phosphate buffer pH 7.4 and was filtered through membrane filter. The aliquot of filtered solution was diluted was further diluted with phosphate buffer pH 7.4 and were analyzed through UV-visible spectrophotometer at 271 nm. The experiment was performed in triplicate and the average was calculated [5].

### 2.3. *In Vitro* Dissolution Studies Protocol

To investigate the release patterns of ciprofloxacin HCl, *in vitro* dissolution studies were performed in phosphate buffer pH 7.4 using Pharma test dissolution apparatus (D-63512, Hainburg). The USP method-I (rotting basket method) was adopted for dissolution studies of ciprofloxacin HCl. One compressed ciprofloxacin HCl 200 mg tablet was added to each station of dissolution apparatus containing 900 mL dissolution solvent. The temperature of the solvent was kept constant at 37°C ± 0.1 and the rotating speed was kept constant at 100 rpm. At predetermined time intervals samples of 5 mL each were withdrawn from the dissolution solvent of each station with the help of special collecting syringe and the dissolution solvent was immediately replaced by fresh solvent already stored at the same temperature. These samples were filtered through membrane filter (0.45 *μ*m) to filter any macroparticle present in the sample. Three replicates of every sample were analyzed for drug concentration using UV-visible spectrophotometer at 271 nm of detection wave length and percentage releases were calculated.

### 2.4. *In Vitro* Drug Release Kinetics

The *in vitro* drug release mechanism of ciprofloxacin HCl from polymeric tablets in phosphate buffer pH 7.4 can be described by fitting the dissolution data in five different kinetic models as given below.

**Zero-order kinetics:**

(1) $$W=K_{1}t$$

(see [6]).

**First-order kinetics:**

(2) $$\mathrm{In}\left(100-W\right)=\ln100-K_{2}t$$

(see [6]).

**Higuchi kinetics:**

(3) $$W=K_{4}t^{1/2}$$

(see [7]).

**Hixson-Crowell kinetics:**

(4) $${\left(100-W\right)}^{1/3}=100^{1/3}-K_{3}t$$

(see [6]).

**Korsmeyer-Peppas kinetics:**

(5) $$\frac{M^{t}}{M\infty }=K_{5}t^{n}$$

(see [8]).

In Korsmeyer-Peppas kinetic model an (*n*) value which is a diffusional exponent represents the mechanism of drug release from matrix tablets. When *n* = 0.5 then drug diffuses with a quasi-Fickian diffusion mechanism from a matrix tablet. When the value of *n* > 0.5 then anomalous or non-Fickian diffusion mechanism of drug occurs and when *n* = 1 then non-Fickian, zero-order or case-II release kinetics occurs.

### 2.5. Stability and Reproducibility Study

In order to determine the reproducibility of the manufacturing process, three different batches of selected formulations from the test were prepared at three different periods. Tablets were packed in tightly air-closed high-density polyethylene jars and a proper accelerated storage conditions was maintained, that is, 40 ± 2°C temperature and 75 ± 5% relative humidity (RH) using a stability chamber (Ti-Sc-THH-07-0400 Faisalabad, Pakistan). These storage conditions were in accordance with international commission for harmonization guidelines for a 6-month period. After the due time these tablets were evaluated for several physicochemical parameters including appearance, hardness, friability, and percent drug content before storage (0 time) and after storage for 1, 2, 4, and 6 months [9].

### 2.6. *In Vivo* Study Protocol

The *in vivo* study in rabbits was approved by the Ethical Committee of Gomal University, Dera Ismail Khan (KPK), Pakistan. For *in vivo* study ciprofloxacin HCl-ETHOCEL CR tablets, an optimized formulation prepared at D : P ratio 10 : 3 was selected. For *in vivo* study, 10 healthy Himalayan angora rabbits were selected and the study calculations were done according to a randomized two-way crossover design. The selected rabbits were having average weight of 2 ± 0.3 kg. These 10 rabbits were divided into two groups (Group A and Group B) each group having 5 rabbits and the study was conducted for two trial periods each having one-week duration.

As shown in Table 2, in Period I the rabbits of Group A were given the tablet from the test formulation and Group B was given ciprofloxacin conventional tablet with trade name of ciproxin by Bayar Pharma as reference standard formulation. Similarly in Period II the rabbits of Group A were given the reference standard formulation and Group B were given the tablets from the test. Each preparation was administered to the rabbits in the morning at 8.00 a.m. after a 24 hr fast. The tablets were introduced into the base of rabbits tongue for ingestion through a plastic needle followed by a few droughts of water nearly 15 mL.

Throughout the study period the rabbits were having free excess to water but were kept fasted for 12 hrs after the dosage administration. At specific time intervals blood samples, each 0.7 mL, were collected from the marginal ear vein of each rabbit and stored in small 3 mL tubes and were allowed to clot. In another 3 mL glass tube, a 200 *μ*L serum was taken and centrifuged at 3000 rpm for 10 min and the plasma from each tube was transferred to new tubes and was kept in refrigerators at −20°C for further studies [10].

### 2.7. HPLC Analysis of Plasma Ciprofloxacin HCl Concentration

A reversed-phase high-performance liquid chromatographic method was used for the quantitative analysis of ciprofloxacin HCl in rabbit blood plasma. This system was comprised of HPLC (Perkin Elmer Series 200, USA) having a TCNav software for operation. This HPLC consists of binary pump solvent delivery system, a UV-visible wavelength detector, and integrator NCI 900. The chromatographic separation was carried out on partition (5 *μ*m pore size), 4.0 mm × 250 mm ODS Hypersil C18 stainless steel analytical column fitted with a refillable guard column. The detector was operated at 271 nm wavelength. The mobile phase for HPLC analysis comprised 75 mM phosphate buffer, methanol, and acetonitrile (ratio 48 : 26 : 26 v/v). The plasma analysis was done at a flow rate of 1.0 mL/min and quantification was done by height of peak. In order to quantify the plasma concentration of ciprofloxacin HCl from the regression equation, the peak height ratio of ciprofloxacin HCl was determined.

Prior to administration of injection, the plasma samples were treated to extract ciprofloxacin HCl by the following procedure: Plasma sample (100 *μ*L) was taken in a glass tube with a screw cap usually made of teflon followed by the addition of 100 *μ*L of 1 M sodium hydroxide and 6 mL mixture of pentane and dichloromethane (ratio 85 : 15). This mixture was vortexed for 1-2 min with the help of vortex mixture and then was centrifuged for 10 min at 3000 rpm. In centrifugation, an upper organic layer was separated which was taken in a reactivial and the solvent was evaporated until the complete dryness of extract under a gentle stream of nitrogen at a constant temperature of 40°C. The final residue was taken in a glass tube and was reconstituted with 100 *μ*L of acetonitrile and mixed for 1 min by vortex mixture, and a 50 *μ*L of this reconstituted solution was injected into the HPLC column with the help of syringe. For the percentage recovery of ciprofloxacin HCl, linearity, precision, and accuracy of the method, blank plasma was taken and spiked with a known amount of ciprofloxacin HCl to give concentrations of 5, 10, 20, 40, 80, and 160 ng/mL and fixed concentrations of ciprofloxacin stock solution (5, 10, 20, 40, 80, and 160 ng/mL).

### 2.8. Analysis of Pharmacokinetic Parameters

Three different pharmacokinetic parameters including plasma concentration time curve (AUC_0-*∞*_), peak plasma concentration (*C*_max⁡_), and time to reach peak plasma concentration (*T*_max⁡_) were estimated from the plasma concentration-time profile of individual rabbit serum in which *C*_max⁡_ and *T*_max⁡_ were calculated directly from the arithmetic plot of blood plasma concentration of ciprofloxacin HCl versus time profile of individual rabbit serum and AUC_0-*∞*_ was estimated by trapezoidal rule. The elimination rate constant *K*_*e*_ was determined from the terminal slope of individual plasma concentration versus time curve after the logarithmic transformation of plasma concentration values and application of linear regression [11]. Similarly, the apparent volume of distribution (*Vd*/*f*) was calculated by the equation: Dose/(AUC_0-*∞*_ × *K*_*e*_) while the elimination half-life (*t*_1/2_) was calculated from the quotient ln⁡⁡2/*K*_*e*_. For the determination of individual absorption profile the following equation was used:

(6) $$\begin{array}{c} \%\;\text{absorbed}\;\text{at}\;\text{time}\;t=C_{t}+\frac{K_{e}{\text{AUC}}_{0\text{-}t}}{K_{e}{\text{AUC}}_{0\text{-}\infty }}, \end{array}$$

where *C*_*t*_ is plasma concentration at time *t*, *K*_*e*_ is the elimination rate constant, AUC_0-*t*_ is the area under the plasma concentration time curve from any time zero to time *t*, and AUC_0-*∞*_ is the total area under the plasma concentration versus time curve.

### 2.9. Statistical Analysis

A statistical procedure, analysis of variance (ANOVA), was used to calculate the values of certain pharmacokinetic parameters such as AUC_0-*∞*_,  *C*_max⁡_,  *t*_1/2_, *K*_*e*_, and *Vd*/*f* obtained with the two preparations. This procedure has a distinguished effect due to subjects, treatments, and periods. The *t*_max⁡_ values of the two preparations were analyzed using the wilcoxon signed rank test for paired samples. Hence, a statistically significant difference was considered when *P* < 0.05.

### 2.10. Relative Bioavailability and *In Vivo-In Vitro* Correlation

The following formula was used to calculate the percent relative bioavailability of the formulations from the test:

(7) $$\begin{array}{c} \text{Percent}\;\text{relative}\;\text{bioavailability}=\frac{{\text{AUC}}_{0\text{-}t}\;\left(\text{test}\right)}{{\text{AUC}}_{0\text{-}t}\;\left(\text{reference}\right)}\times 100. \end{array}$$

For *in vivo*-*in vitro* correlation, an optimized formulation (10 : 3) was selected and investigated by plotting *P*_*a*_ (percent drug absorbed) against *P*_*r*_ (percent drug released). Wagner-Nelson method [12] was used to calculate percent drug absorbed values while the *in vitro* drug release data was used to calculate percent drug released data [9].

## 3. Results

The present study was conducted to formulate and evaluate controlled release drug delivery system for ciprofloxacin HCl using ETHOCEL 7P as matrix material. The results of different physicochemical parameters applied to CR formulations of ciprofloxacin HCl could be seen in Table 3. These parameters were evaluated to assess the quality of tablets and also serve as a pointer to good manufacturing practices (GMP). The tablets were having good smooth surfaces and elegant in appearance. Weight is compendial standard to assess the quality of tablets, in case of ciprofloxacin HCl controlled release matrix tablets the weight variation ranges between 199.8 mg to 203.2 mg which was in USP acceptable range (>0.5%) which indicate good uniformity of weight (5). The hardness of the tablets was in the range from 6.4 ± 0.12 to 7.1 ± 0.18 which was within acceptable range (5–10 kg/cm^2^). The thickness of the CR tablets was not more than 2.8 ± 0.08 mm which were in range (2–4 mm). The diameter of all the tablets was 8 ± 0.07 mm which was also with in USP acceptable limits (4–13 mm). The loss of weight of tablets ranges from 0.27 ± 0.09% to 0.39 ± 0.22% which was in limits (<0.8%). The drug content of CR tablets of all the formulations ranges from 97.5 to 99.1 which shows a good content uniformity of drug.

### 3.1. *In Vitro* Drug Dissolution and Kinetics

**Figure 1:**
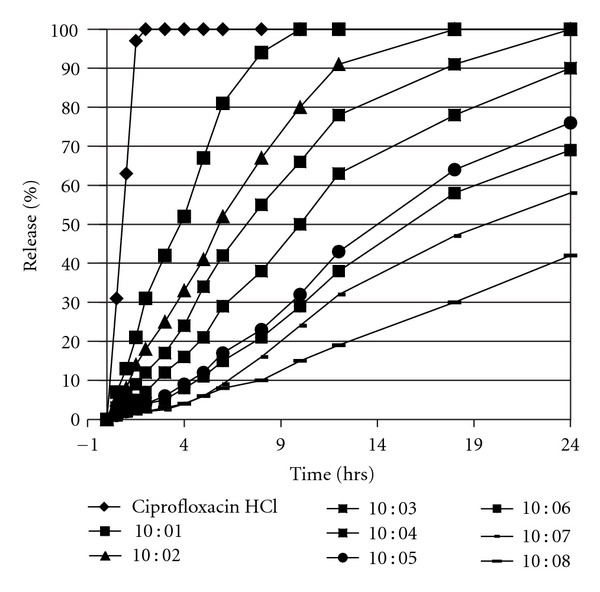
*In vitro* release profiles of ciprofloxacin HCl controlled release tablets prepared at different D : P ratios in phosphate buffer pH 7.4 (mean ± SEM, *n* = 6).

Figure 1 shows the *in vitro* drug release profiles of ciprofloxacin HCl from polymeric tablets prepared at eight different drug-to-polymer ratios with respect to time. In all the developed formulations, the formulation prepared at D : P ratio of 10 : 3 gave the expected result by releasing about 99% drug in 24 hrs. It could be mentionable that the hardness of the tablets did not significantly affect the drug release rates.

Zero-order kinetic model was found to best describe the *R* ^2^ (coefficient of determination). Korsmeyer-Peppas equation also best suits the dissolution data where the values of “*n*” were >0.9 indicating anomalous, non-Fickian, or nearly zero-order release mechanism [13].

### 3.2. Stability and Reproducibility of Manufacturing Process

**Figure 2:**
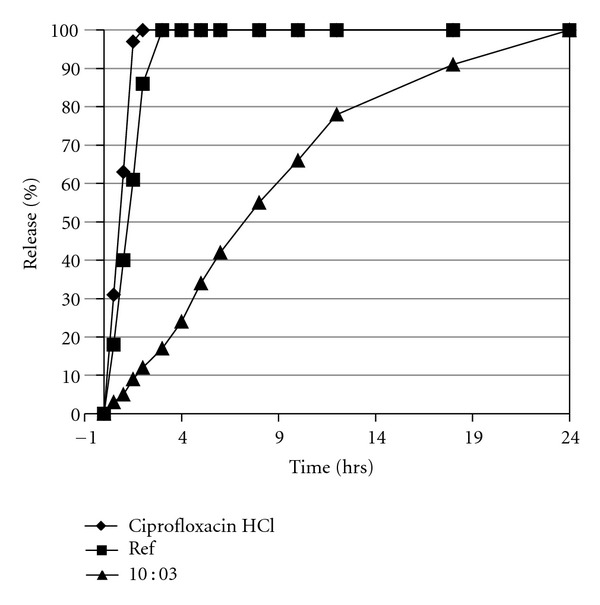
Comparative release profiles of ciprofloxacin HCl, a reference standard and test CR formulation (D : P ratio 10 : 3) in phosphate buffer pH 7.4 (mean ± SEM, *n* = 6).

The formulation with good dissolution profile, that is, prepared at D : P ratio 10 : 3 was selected as an optimized formulation with 7.4 ± 0.3 kg/cm2 hardness, 0.27 ± 0.11% friability, and 24 hrs drug release profile (Figure 2). This formulation exhibit nearly zero-order release profile with (*n* = 0.933) in dissolution medium. As shown in Table 4, in all the three batches of these tablets no significant difference (*P* < 0.05) was observed with respect to drug content. Several parameters were evaluated for the stability study including hardness, friability, drug content, weight variation, and physical appearance showing no significant difference at accelerated storage conditions (40°C ± 2 & 75 ± 5% RH) after storage for 1, 2, 4, and 6 months.

### 3.3. *In Vivo* Drug Performance

*In vivo* drug release performance was done in rabbit serum by using high-performance liquid chromatography. During *in vivo* study the rabbit serum collected at zero time showed no peak while the serum samples spiked with ciprofloxacin HCl showed single peaks of the drug indicating that the adopted method was highly selective.

**Figure 3:**
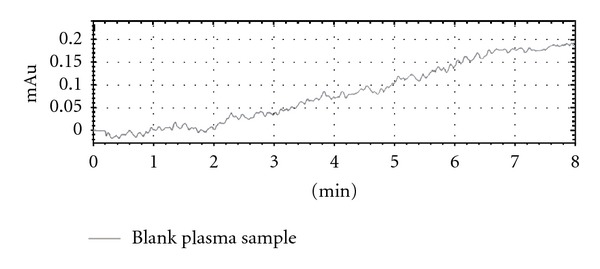
HPLC chromatogram of blank plasma.

**Figure 4:**
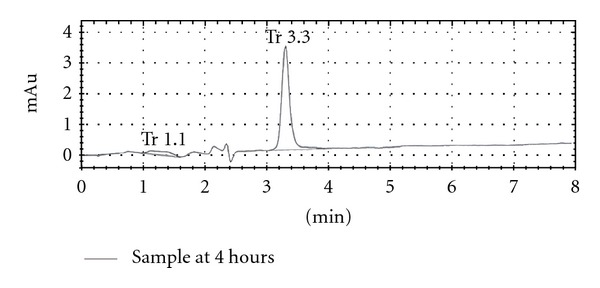
HPLC chromatogram of sample obtained from rabbit serum at 4 hours after drug administration.

The following Figures 3 and 4 shows the HPLC chromatograms of blank plasma and plasma sample of rabbit serum after 4 hrs of test formulation administration containing ciprofloxacin HCl. The retention time of ciprofloxacin HCl was 3.30 min with no interfering peak at the retention time of ciprofloxacin HCl while the blank sample was clean.

The absolute recovery and retention time for ciprofloxacin HCl was 95%. The coefficient of determination (0.901) showed a good level linearity of the method.

### 3.4. Pharmacokinetics of Ciprofloxacin HCl

Ciprofloxacin HCl controlled release tablets showed significantly different *T*_max⁡_ (8 ± 2.7 hrs) as compared to *T*_max⁡_ of reference standard (4 ± 1.2 hrs), where *P* < 0.05. Significantly higher values of peak time were observed. The half-life *t*_1/2_ of test CR formulation was observed to be 18 ± 1.97 hrs as compared to reference conventional tablet 8 ± 0.78 hrs, where *P* < 0.05. The higher values of *T*_max⁡_ and half-life *t*_1/2_ for test CR formulations indicate the extended absorption phase and the presence of drug for a long time in the body. Moreover, significantly optimized values of peak concentration (*C*_max⁡_) were observed for test CR tablets (44.7 ± 1.34 ng/mL) as compared to reference conventional formulation (78.32 ± 2.19 ng/mL), where *P* < 0.0001. Similarly, AUC_0-*t*_ for test formulation was 2251 ± 19 ng·hr/mL as compared to reference standard conventional formulation being 1962 ± 24 ng·hr/mL and AUC_0-*∞*_ was 2157 ± 27 ng·hr/mL and 2297 ± 21 ng·hr/mL for test formulation and reference standard, respectively, which were not significantly different having *P* < 0.05. Nearly similar values of *C*_max⁡_,  AUC_0-*t*_, and AUC_0-*∞*_ indicate bioequivalence of the test CR formulations to the reference standard conventional tablets.

### 3.5. *In Vivo-In Vitro* Correlation and Relative Bioavailability

The test tablets were evaluated for relative bioavailability and were found to 95% with a significant extended absorption phase.

As shown in Figure 6, by plotting the percent drug released (*in vitro*) against percent drug absorbed (*in vivo*) the value of *R* ^2^ was 0.934 indicating a good *in vivo-in vitro *correlation.

## 4. Discussions

During *in vitro* dissolution studies, it was observed that as compared to reference standard, the drug release patterns of all the test formulations extend to a longer time but most optimized was the formulation prepared at D : P ratio 10 : 3. During the *in vitro* dissolution, a common phenomenon of tablet swelling was observed which might be due to the hydration of the polymer. This swelling leads to rapid decrease in glass transition temperature (*T*_*g*_) of the polymer to the temperature of the dissolution solvent. The dissolution solvent exerts a stress on the polymer due to which a relaxation response occurs in polymer chains which increase the distance among the polymer chains leading to swelling of the tablet [14]. Moreover, due to hydration the volume of polymer increases which reduce the free volume because of the formation of microspores which itself acts as a shift in drug transport mechanisms [15]. From the *in vitro* drug release profiles it could be observed that the release was highly dependent on polymer concentration, as the amount of polymer was increased more extended release profiles were obtained and a linear curve was obtained shifting the drug transport mechanism to nearly zero order or zero-order release kinetics. The reduction in drug release rates and extended release profiles by increasing the polymer concentration may be due to slow hydration of the matrix material depending upon the hydrophobic character of ETHOCEL [9]. It is suggested that on dehydration the matrix material swallows and enclosed the drug thus allowing a small amount of drug in certain time through small microspores of the swollen tablet [4, 15]. Similar results were found while studying the effect of polymers on *in vitro* drug release profiles of ibuprofen, where the increase in concentration of polymer decreases the drug release and exhibit extended drug release profiles [16]. The selection of ETHOCEL was based on the fact that ETHOCEL is a hydrophobic polymer so is not significantly affected by the variable pH of the GIT thus could give ciprofloxacin release from the polymeric tablet irrespective of the change of pH of GIT [17].

Drug release mechanism from ciprofloxacin controlled release dosage forms was elucidated by fitting the *in vitro* dissolution data in Korsmeyer-Peppas equation which best describes the drug transport mechanism where the value of diffusion coefficient “*n*” was calculated through the slope of the straight line of the data. The value of “*n*” for the optimized formulation was greater than 0.5 and lesser than 1 (1 > *n* > 0.5) indicating nearly zero-order, anomalous, or non-Fickian case II transport mechanism, where Fickian is drug transport through pores of tablet matrix, zero order indicates a drug transport mechanism through the erosion of polymer chains, and anomalous demonstrates the drug transport mechanism through a combined effect of diffusion and erosion [9]. The linearity of the regression line was determined by coefficient of determination *R* ^2^ the value of which was nearly 1.

In the presented study, animal models were used to study the *in vivo* performance of ciprofloxacin HCl which showed that the developed CR formulations maintain a constant blood plasma level for approximately long period of time as compared to a reference conventional dosage form which gain a rapid peak plasma concentration but could not maintain it. The extended half-life *t*_1/2_ and the time which is required to achieve peak plasma concentration *T*_max⁡_ were representative of a slower drug release rate and an extended time period. The AUCs of both the test and reference formulations were not significantly different indicating that the formulations were bioequivalent. ETHOCEL were found a significant rate controlling polymer which can produce tablets of desired hardness and friability giving a nearly zero-order drug release and maintaining a nearly constant peak plasma level in desired therapeutic rang thus minimizing the risk of reaching drug plasma concentrations above MTC (maximum toxic concentration), therefore, avoiding drug toxicity and side effects and improves tolerability and adherence [9].

**Figure 5:**
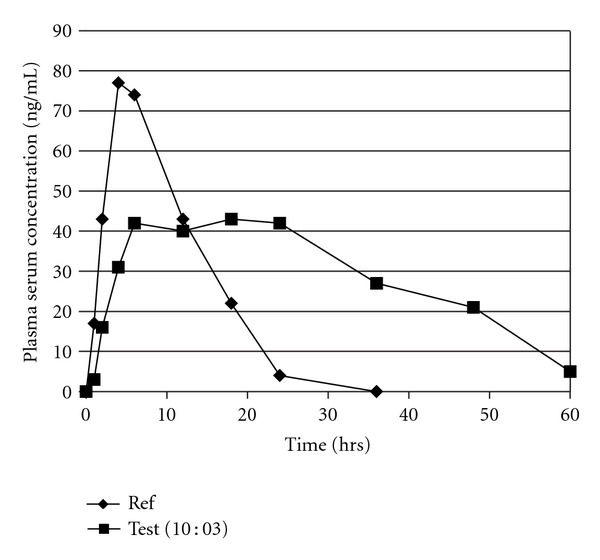
Comparative plasma concentrations versus time profiles of ciprofloxacin HCl test tablets and reference standard conventional tablets (mean ± SEM, *n* = 6).

**Figure 6:**
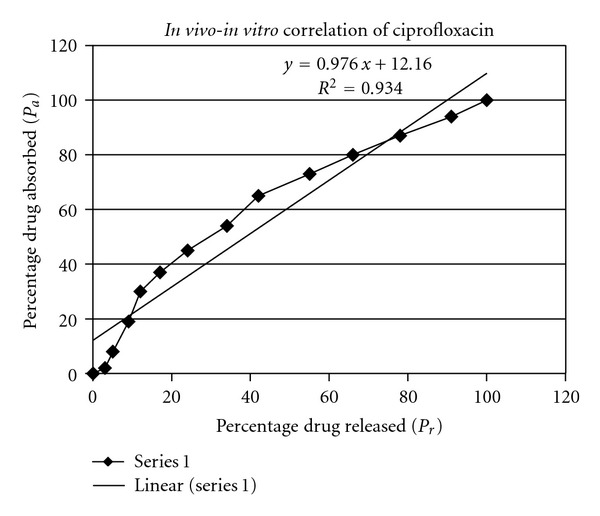
Percent drug released (*P*_*r*_) plotted against percent drug absorbed (*P*_*a*_) of ciprofloxacin HCl.

The plasma serum concentration of ciprofloxacin HCl versus time profile from reference conventional formulation and test formulation could be seen from the Figure 5 given above. It could be seen that plasma concentrations of ciprofloxacin were detectable even at 60 hr. A gradual increase in serum concentration was observed reaching the peak plasma concentration at about 8 hrs after drug administration via oral route and maintained for about 20 hrs reflecting a controlled release formulation. It was observed that the reference formulation had a higher and faster peak plasma concentration in short time as compared to test CR formulation indicating a slow and steady rate of drug absorption from the CR test formulation. A similar result was found by investigating Olanzapine in rabbit serum after administering a test CR formulation and a reference conventional formulation [9].

Ciprofloxacin HCl controlled release matrix tablets were also evaluated for relative bioavailability and was found to be 95% indicating 95% relative bioavailability for both the test formulation and reference formulation but the test formulation having a stable and constant peak plasma concentration for an extended period of time as compared to the reference conventional dosage form.

A good understanding of *in vivo-in vitro* (IVIVC) correlation being the major objective in preparations of pharmaceutical dosage forms. Dissolution specifications can be established using IVIVCs and can be used for the validation of these dissolution specifications. IVIVCs are widely practiced in the development of oral controlled release/sustain release dosage forms. For *in vivo-in vitro* correlation, the value of coefficient determination *R* ^2^ (0.934) indicating a good correlation between *in vitro* drug release and *in vivo* drug absorption.

## Figures and Tables

**Table 1:** Formulation of 200 mg ciprofloxacin HCl tablets.

Drug	D : P ratio	Polymer	Filler (lactose)	Lubricant (0.5%)
Ciprofloxacin HCl-ETHOCEL CR tablets				
100 mg	10 : 01	10 mg	89 mg	1 mg
100 mg	10 : 02	20 mg	79 mg	1 mg
100 mg	10 : 03	30 mg	69 mg	1 mg
100 mg	10 : 04	40 mg	59 mg	1 mg
100 mg	10 : 05	50 mg	49 mg	1 mg
100 mg	10 : 06	60 mg	39 mg	1 mg
100 mg	10 : 07	70 mg	29 mg	1 mg
100 mg	10 : 08	80 mg	19 mg	1 mg

**Table 2:** *In vivo* drug dosing schedule of rabbits for a two week period.

Group	Period	
	I	II
A	Test formulation	Ref: Ciproxin tabs
B	Ref: Ciproxin tabs	Test formulation

**Table 3:** Values of physicochemical parameters applied to CR tablets of ciprofloxacin HCl.

S/no.	Parameter	10 : 1	10 : 2	10 : 3	10 : 4	10 : 5	10 : 6	10 : 7	10 : 8
(1)	Weight variation (mg)	202.3	201.7	199.8	202.4	200.9	201.3	203.4	203.2
(2)	Friability (%)	0.31 ± 0.17	0.38 ± 0.21	0.34 ± 0.19	0.27 ± 0.09	0.39 ± 0.22	0.22 ± 0.07	0.17 ± 0.04	0.38 ± 0.21
(3)	Hardness (kg/cm^2^)	6.7 ± 0.13	6.4 ± 0.12	6.9 ± 0.16	6.8 ± 0.15	7.1 ± 0.18	6.9 ± 0.13	7.0 ± 0.17	6.8 ± 0.15
(4)	Diameter (mm)	8 ± 0.07	8 ± 0.05	8 ± 0.07	8 ± 0.07	8 ± 0.04	8 ± 0.06	8 ± 0.08	8 ± 0.07
(5)	Thickness (mm)	2.7 ± 0.09	2.7 ± 0.09	2.8 ± 0.08	2.6 ± 0.05	2.6 ± 0.06	2.7 ± 0.08	2.7 ± 0.06	2.5 ± 0.05
(6)	Drug content (%)	98.3	97.8	102.1	97.5	98.9	101.3	99.5	98.7

**Table 4:** Stability parameters of ciprofloxacin HCl CR tablets prepared at D : P ratio 10 : 3 (Mean ± SEM, *n* = 6).

Periods of sampling	Hardness (kg)	Friability (%)	Appearance (colour)	Drug content (%)	Weight variation (%)
Before storage (0 time)	7.4 ± 0.3	0.27 ± 0.11	White	103 ± 2	4 ± 0.3
After 1 month	7.2 ± 0.4	0.19 ± 0.13	White	102 ± 2	5 ± 0.2
After 2 month	7.3 ± 0.2	0.27 ± 0.13	White	103 ± 3	5 ± 0.4
After 4 month	7.1 ± 0.4	0.22 ± 0.11	White	101 ± 2	5 ± 0.3
After 6 month	7.4 ± 0.2	0.24 ± 0.12	White	102 ± 3	4 ± 0.2
